# A window into diaphragm kinetics: feasibility, precision, and physiological meaning of ultrasound measurements of diaphragm thickness

**DOI:** 10.1186/cc12083

**Published:** 2013-03-19

**Authors:** EC Goligher, F Laghi, ND Ferguson

**Affiliations:** 1 University of Toronto, Canada; 2 Loyola University, Chicago, IL, USA

## Introduction

Inadequate respiratory muscle activity has been linked to ventilator-induced diaphragm dysfunction. Current techniques for monitoring respiratory muscle activity during mechanical ventilation are specialized and relatively invasive. Visualizing diaphragm thickening during inspiration by ultrasound may permit non-invasive monitoring. We explored the feasibility, reliability and physiological significance of diaphragm thickening on ultrasound.

## Methods

Five healthy subjects participated. We monitored inspiratory flow, volume, esophageal and gastric pressures, and diaphragm electrical activity (by esophageal and surface electromyography) while subjects performed a series of inspiratory maneuvers: tidal breathing, threshold-loaded breathing, a Muller maneuver, and inspiration to various lung volumes above functional residual capacity. At the end of each inspiratory effort, subjects were instructed to close the glottis and relax the respiratory muscles (so as to maintain lung volume while eliminating diaphragm activation). Sonographic images of diaphragm thickening during these maneuvers were obtained using M-mode with a 13 MHz linear array probe placed in the right ninth, 10th, or 11th intercostal space between the middle and anterior axillary lines.

## Results

Diaphragm thickening in the zone of apposition was readily visualized by ultrasound in all five subjects. Mean end-expiratory diaphragm thickness was 2.1 mm (SD = 0.3 mm). During tidal breathing, the diaphragm thickened by a mean of 35% (SD = 31%). The Bland-Altman coefficient of reproducibility was 0.5 mm; approximately 50% of measurement variability arose from caliper positioning on the ultrasound machine; diaphragm thickness measurements changed as the probe was placed in different intercostal interspaces. Diaphragm inspiratory thickening increased significantly with increasing inspiratory effort but also varied with lung volume independent of effort. At inspiratory volumes below 40% of inspiratory capacity, lung volume change contributed minimally to diaphragm thickening.

## Conclusion

Visualizing diaphragm thickening in the zone of apposition by ultrasound provides a feasible non-invasive technique for monitoring diaphragm activation in healthy subjects. Diaphragm thickening primarily reflects muscular effort rather than altered muscle conformation induced by changes in lung volume, especially at lower inspiratory volumes.

